# Severe erosive lesion of the glenoid in gouty shoulder arthritis: a case report and review of the literature

**DOI:** 10.1186/s12891-021-04217-5

**Published:** 2021-04-12

**Authors:** Huricha Bao, Yansong Qi, Baogang Wei, Bingxian Ma, Yongxiang Wang, Yongsheng Xu

**Affiliations:** Department of Orthopedics, Inner Mongolia People’s Hospital, No. 20 Zhao Wu Da Street, Inner Mongolia Autonomous Region 010017 Hohhot, China

**Keywords:** Shoulder, Glenoid, Gout, Case report

## Abstract

**Background:**

Gout is a metabolic disease characterized by recurrent episodes of acute arthritis. Gout has been reported in many locations but is rarely localized in the shoulder joint. We describe a rare case of gouty arthritis involving bilateral shoulder joints and leading to severe destructive changes in the right shoulder glenoid.

**Case presentation:**

A 62-year-old male was referred for pain and weakness in the right shoulder joint for two years, and the pain had increased in severity over the course of approximately nine months. A clinical examination revealed gout nodules on both feet and elbows. A laboratory examination showed a high erythrocyte sedimentation rate (ESR), high levels of C-reactive protein and hyperuricemia, and an imaging examination showed severe osteolytic destruction of the right shoulder glenoid and posterior humeral head subluxation. In addition, the left humeral head was involved and had a lytic lesion. Because a definite diagnosis could not be made for this patient, a right shoulder biopsy was performed. The pathological examination of the specimen revealed uric acid crystal deposits and granulomatous inflammation surrounding the deposits. After excluding infectious and neoplastic diseases, the patient was finally diagnosed with gouty shoulder arthritis.

**Conclusions:**

Gout affecting the bilateral shoulder joints is exceedingly uncommon, and to our knowledge, severe erosion of the glenoid has not been previously reported. When severe erosion is present, physicians and orthopedic surgeons should consider gouty shoulder arthritis according to previous medical history and clinical manifestations.

## Background

Gouty arthritis is usually monoarticular and frequently involves the synovial joints of the feet and hands and, rarely, the shoulders [1–3]. Monosodium urate crystals produce an inflammatory response that generally results in swollen, tender, hot joints. As described in the literature, the manifestations of shoulder gout are tophaceous deposits in the rotator cuff, intraosseous tophi in the humeral head, and tophi in the bursa around the shoulder joint [4, 5]. Here, we report a rare case of gout involving bilateral shoulder joints that caused severe erosion of the right shoulder glenoid. In the clinical diagnosis and treatment process, gout may be misdiagnosed as degenerative, infectious arthritis or a malignancy, resulting in delays in diagnosis and treatment. To date, only four cases of shoulder gout have been reported in the English-language literature identified in PubMed, and we provide a brief literature review concerning shoulder gout [4–7]. The patient was informed that data concerning the case would be submitted for publication, and he provided consent.

## Case presentation

A right-hand-dominant, 62-year-old obese man presented to our department due to progressive right shoulder pain and weakness. There was no history of recent trauma to the right shoulder. He had a 2-year history of intermittent pain in the right shoulder. Nine months prior, he started to experience worsening pain and weakness in the right shoulder with the restriction of active shoulder motion. He had been treated with conservative treatment (acupuncture, physical therapy, and subacromial steroid injections), which provided short periods of relief. The pain did not disappear, and he visited our hospital for further examination and treatment.

His past medical history included right clavicle fracture, hypertension, and gout. Thirty-six years prior, he suffered a right clavicle middle-shaft fracture that was treated conservatively with a figure-of-eight bandage. The patient recalled that there was no abnormality in the right shoulder at that time. He had a 20-year history of long-standing but suboptimally treated gout, and the gout intermittently led to redness and pain in the feet, which occurred 4–5 times a year. The symptoms were relieved by colchicine during acute episodes, and no systemic treatment was given. The patient had a body mass index of 31.9 kg/m^2^ and no previous history of tuberculosis. The patient consumed excessive alcohol and had an alcohol consumption history of 250 ml/day for 30 years; additionally, he smoked 20 cigarettes a day for 35 years and followed no particular diet.

**Fig. 1:**
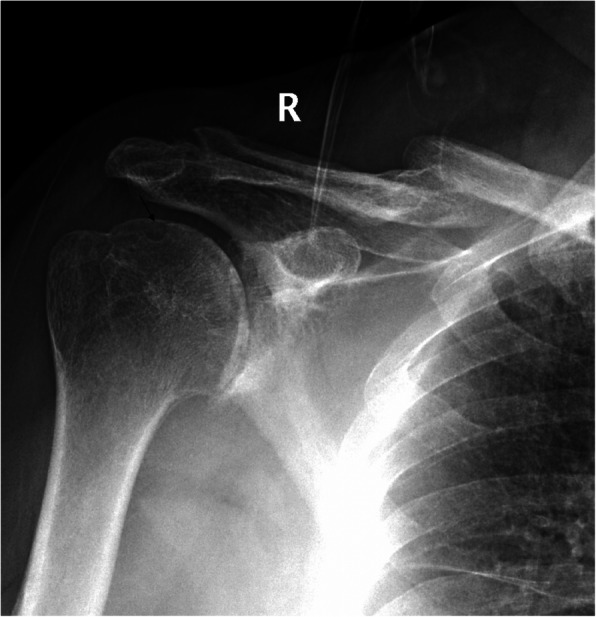
Plain radiographs of the right shoulder showed glenohumeral joint space narrowing and osteophyte formation on the inferior aspect of the glenoid. At the superolateral point of the humeral head, a lytic lesion (arrow) was detected, and malunion of the right clavicle fracture was also seen

**Fig. 2:**
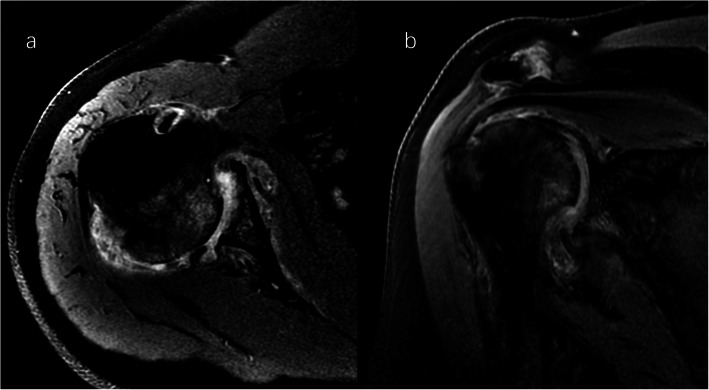
Right shoulder magnetic resonance imaging (proton density-weighted fat-suppressed sequence ). Axial (**a**) and coronal (**b**) views revealed intact rotator cuff, joint effusion, synovial proliferation, effusion within the biceps long head tendon sheath, humoral head superolateral cystic erosion, posterior humeral head subluxation, and severe glenoid erosion

A clinical evaluation revealed that his right shoulder joint had a limited active range of motion, and the passive range of motion was nearly normal. On palpation, tenderness was noted in the anterior and posterior aspects of the shoulder joint, and there was no warmth, erythema, swelling, or redness. When the patient’s upper limb was raised, abducted, and externally rotated, the shoulder joint had a sense of movement, and there were a popping sound and a feeling of shoulder reduction. Gouty tophi were observed on the dorsal aspect of the bilateral great toe and extensor aspect of the bilateral elbows; all of the patient’s other joints were clinically normal, and the examination revealed nothing else of note. Plain radiographs of the affected shoulder showed glenohumeral joint space narrowing, erosions of the glenoid, and osteophyte formation on the inferior aspect of the glenoid. At the superolateral point of the humeral head, a lytic lesion (arrow) was detected, and malunion of the right clavicle fracture was also seen (Fig. 1). To assess the integrity of the soft tissue of the shoulder joint, we ordered a magnetic resonance imaging (MRI) examination, which revealed that the axial and coronal proton density-weighted, fat-suppressed MRI exhibited an intact rotator cuff, joint effusion, synovial proliferation, effusion within the biceps long head tendon sheath, humoral head superolateral cystic erosion, posterior humeral head subluxation, and severe glenoid erosion (Fig. 2). A laboratory examination revealed an elevated uric acid level of 594 µmol/L (normal range 208.00-428.00 µmol/L), erythrocyte sedimentation rate (ESR) of 65 mm/h, C-reactive protein level of 34 mg/L, leukocyte count of 8.35 × 10^9^/L, and hemoglobin level of 12.8 g/dL. The patient had a negative test for rheumatoid factor and anti-citrullinated protein antibodies (ACPAs). The liver and kidney function of the patient were normal. No abnormalities were found on electromyography of the upper extremities. In consideration of the possibility of shoulder joint infection or malignancy, arthrocentesis was performed, and 20 ml of fluid was aspirated. The fluid was macroscopically cloudy and yellow. The synovial analysis revealed an inflammatory cell count with leukocytes 5200/mm^3^, which were predominantly neutrophils. Gram staining of the fluid was negative, and no organisms were cultured. A cytology analysis and the joint fluid Xpert MTB/RIF test were negative. A polarizing microscope was not available in our hospital; therefore, we could not examine the synovial fluid for crystals.

**Fig. 3:**
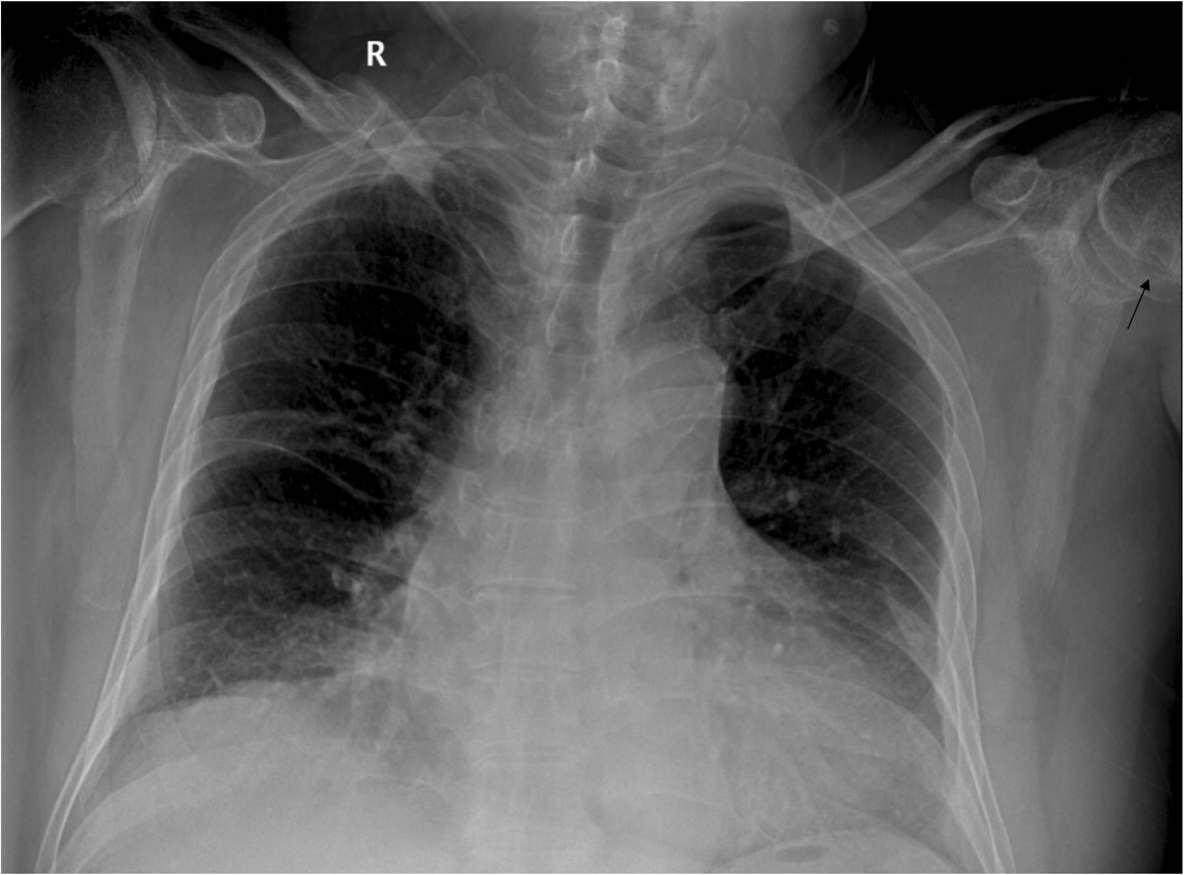
The plain radiograph of the chest showed no neoplastic or tuberculous changes. Serendipitously, the radiograph revealed a round contour lytic lesion (arrow) in the left humeral head with sclerotic borders near the articular surface

The plain radiograph of the chest showed no neoplastic or tuberculous changes. Serendipitously, the radiograph revealed a round contour lytic lesion in the left humeral head with sclerotic borders near the articular surface (Fig. 3). Therefore, a bilateral shoulder joint computed tomography (CT) scan and left shoulder MRI examination were performed. An infectious shoulder etiology appeared unlikely, and differential diagnoses included destructive arthritis or neoplastic lesions. Ultrasound and dual-energy CT imaging of the shoulder joints were not performed on this patient.

**Fig. 4:**
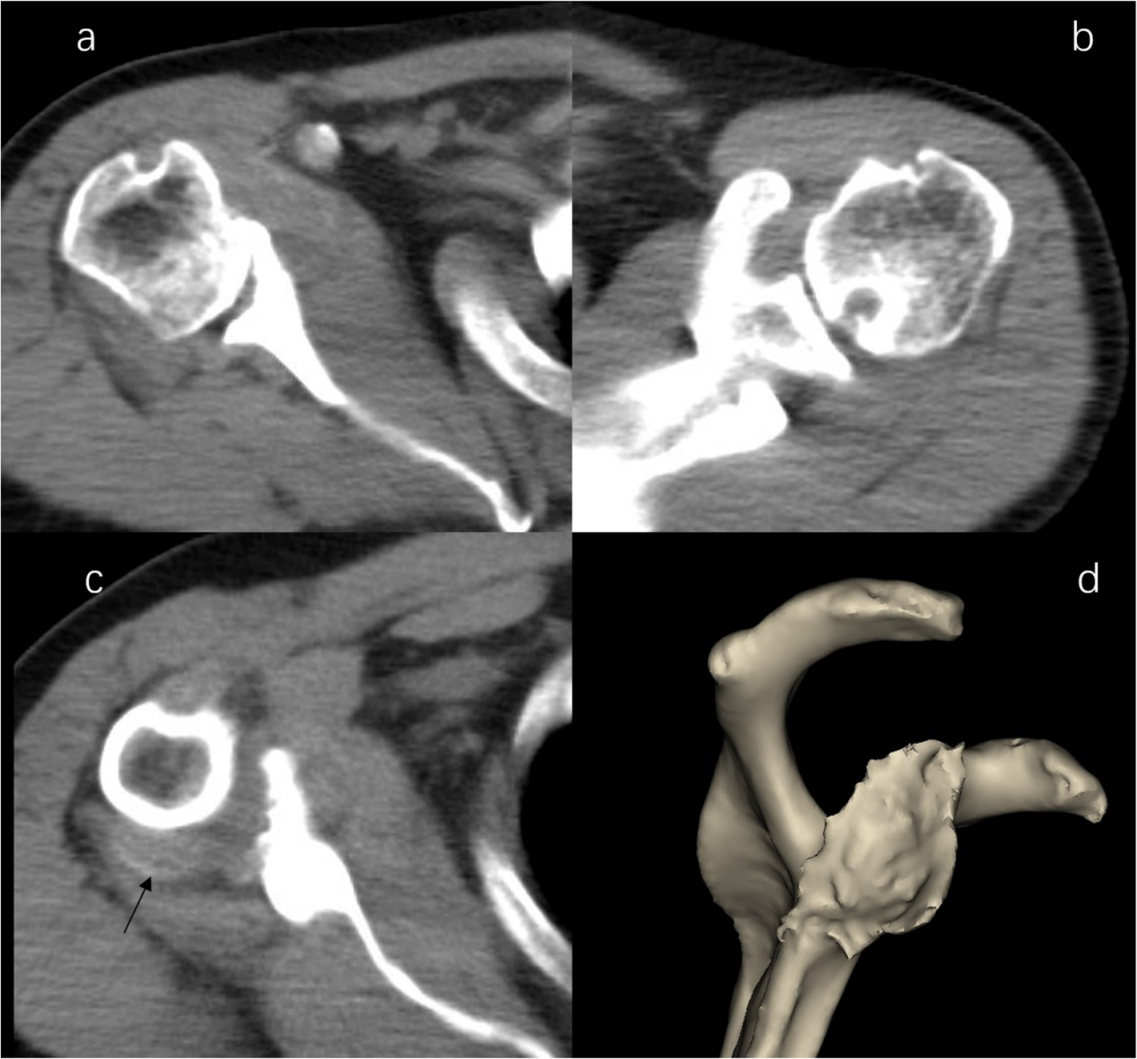
CT image of shoulder. b Right shoulder CT image demonstrated severe destructive lytic changes at the glenoid and erosive lesions and posterior subluxation of the humeral head in the right shoulder. **b** Left shoulder CT image shows a circular lytic lesion in the left humeral head with sclerotic borders near the articular surface broke through the articular cartilage. **c** a faint amorphous opacity (arrow) be seen at the posterior capsule of right shoulder. **d** 3D image shows severe destructive defect of the right shoulder glenoid

**Fig. 5:**
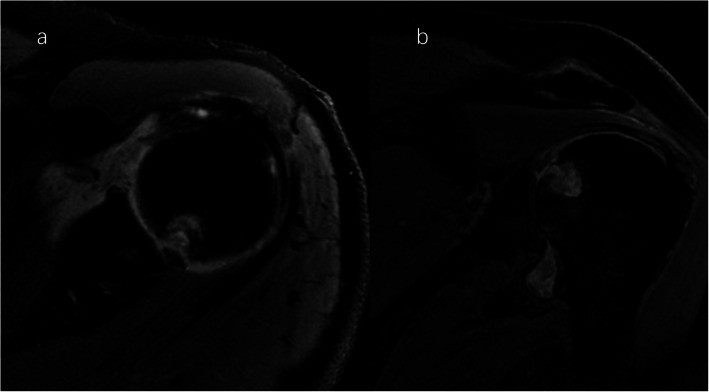
Light shoulder magnetic resonance imaging (proton density-weighted fat-suppressed sequence ). Axial (**a**) and coronal (**b**) MRI image demonstrate effusion, cystic destruction of the subchondral bone of the humeral head, erosion, and collapse of articular cartilage medial to the lesion

**Fig. 6:**
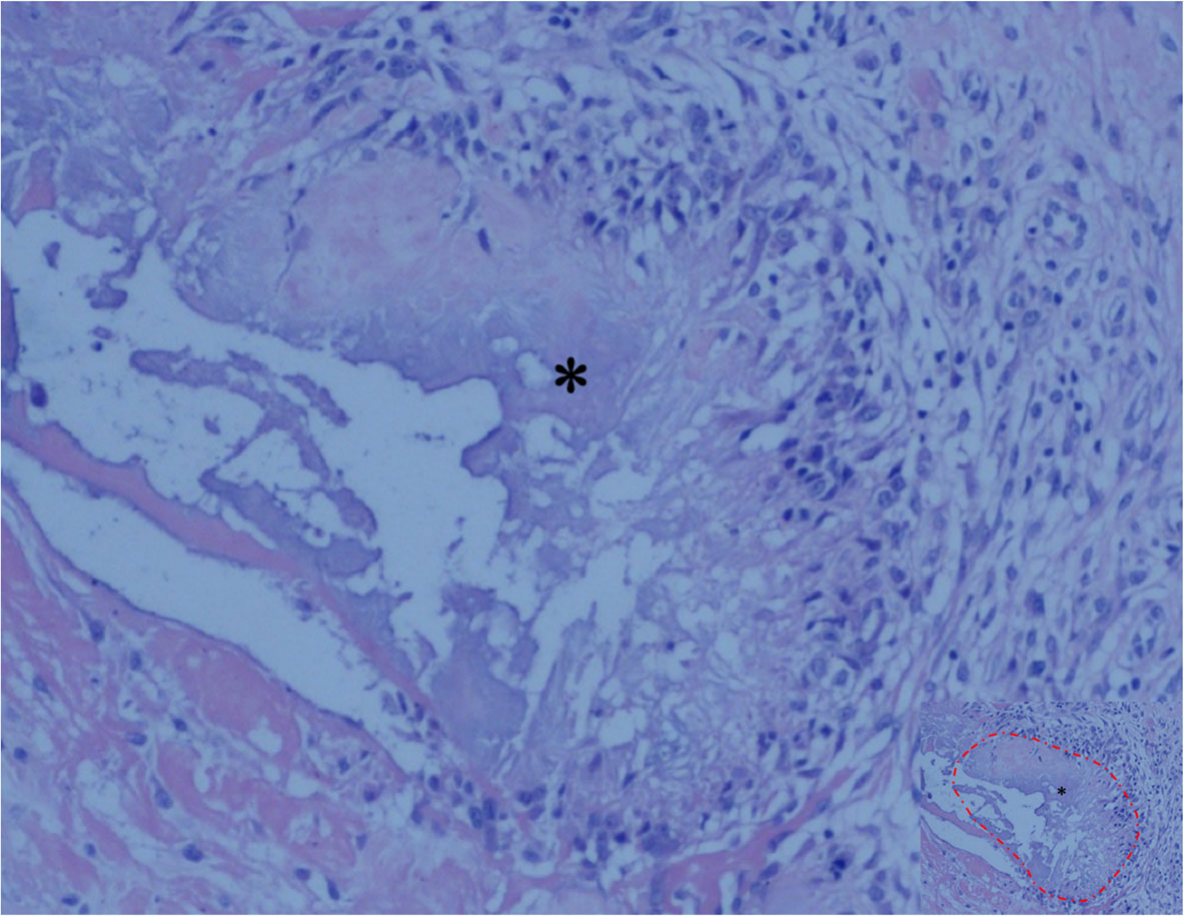
Tissue specimen from the right shoulder showing the deposition of uric acid crystals (asterisk, the part marked with a red dashed line in image with reduced size) surrounded by granulomatous inflammation, and there was no evidence of malignancy or tuberculosis (hematoxylin and eosin; original magnification, ×100)

The CT scan demonstrated severe destructive lytic changes at the glenoid and erosive lesions and posterior subluxation of the humeral head in the right shoulder. A faint amorphous opacity could also be seen at the posterior capsule of the right shoulder. Circular lytic lesions in the left humeral head with sclerotic borders near the articular surface broke through the articular cartilage (Fig. 4). Left shoulder MRI revealed that the axial and coronal proton density-weighted, fat-suppressed MRI showed effusion, lytic destruction of the subchondral bone of the humeral head, erosion, and collapse of the articular cartilage medial to the lesion (Fig. 5). However, we still could not determine the cause of the bony destruction of the shoulder joint. Therefore, several biopsies were taken from the capsule and synovial membrane of the right shoulder. The biopsy showed inflammatory cells and gout crystals, and there was no evidence of malignancy or tuberculosis (Fig. 6). Based on these findings, we made a diagnosis of gouty arthritis of the bilateral shoulder. Etoricoxib and febuxostat treatment improved the patient’s clinical condition. The patient refused surgical intervention and decided to continue receiving physical therapy and medication for symptom control. After physical therapy and medication, the pain in the right shoulder diminished further but was not eliminated.

## Discussion and conclusions

Gout is a common inflammatory joint disease characterized by monosodium urate crystal deposition in joints and connective tissue. Gout often affects the feet, hands, elbows, and knees and is relatively uncommon in the shoulder joint. We presented a rare case of gouty arthritis involving bilateral shoulder joints and leading to severe destructive changes in the right shoulder glenoid.

The clinical stage of gout is divided into asymptomatic hyperuricemia, acute gouty arthritis, the intercritical period, and chronic tophaceous gout [8]. The typical clinical manifestation of acute gouty arthritis is characterized by sudden, severe pain, swelling, redness, and tenderness in the joints. Poorly controlled gout may develop into chronic tophaceous gout, and long-standing gout may present with more atypical symptoms. A chronic deposition of monosodium urate crystals in joints and other body tissues can manifest as a wide array of presentations and can lead to severe joint damage. Such atypical presentations are likely a result of the complexity of reasons. In our case, the patient’s symptoms were pain and weakness in the right shoulder, and the onset of symptoms was relatively insidious, while the patient’s left shoulder joint did not have any clinical symptoms or discomfort. A plain radiograph of the patient’s right shoulder joint revealed degenerative changes, including glenohumeral joint space narrowing, erosion, and osteophyte formation on the glenoid, with a lytic lesion on the humeral head. The left shoulder showed an osteolytic lesion of the humeral head. MRI revealed an intact rotator cuff, inflammatory synovitis, humoral head cystic erosion, posterior humerus subluxation, and a severe glenoid defect. The CT scan further confirmed the severe bone erosive defect of the right shoulder glenoid and posterior humeral head subluxation. From the CT scan, we also observed a round contour lytic lesion with sclerotic borders near the articular surface in the left humeral head. We did not find obvious intra- or extra-articular gout crystal deposition on plain radiographs, CT scans, or MRI images of the bilateral shoulder joints, except in the bilateral humeral head and at the posterior capsule of the right shoulder.

Radiological characteristics of chronic gouty arthritis include punched-out erosions with overhanging cortex and sclerotic margins, preservation of joint space, and dense nodules of soft tissues, which are sometimes calcified [9]. The typical MRI manifestations of tophaceous gouty arthritis are homogeneous intermediate signal intensity on T1-weighted images and heterogeneous intermediate-to-low signal intensity on T2-weighted images, depending on the calcium concentration within a tophus [10]. There are several descriptions of gouty shoulder arthritis in the literature, as well as rare cases of coexisting gout and septic shoulder arthritis. Sean et al. first reported a case of subacromial impingement caused by tophaceous gout of the rotator cuff. There was no abnormality on MRI except supraspinatus tendonitis. Arthroscopic findings revealed tophaceous deposits of the supraspinatus and subscapularis tendon [4]. Chao et al. reported a case of tophaceous gout involving the rotator cuff. In this case, the patient complained of intermittent pain and a limited range of motion of the right shoulder after a shoulder injury. A plain radiograph of the right shoulder demonstrated a faint amorphous opacity above the humeral head. MRI revealed urate crystal deposits in intrasubstance areas and the articular side of the supraspinatus tendon [5]. Toru et al. reported the coexistence of gouty and septic shoulder arthritis after arthroscopic rotator cuff repair surgery. The patient developed a high fever postoperatively, and at the same time, the left shoulder presented pain, swelling, and warmth. However, the article did not describe the imaging manifestations of the shoulder joint [6]. Ana et al. reported a case of tophaceous gout of the right shoulder joint. The patient presented with pain in the right shoulder with certain movements and when lying on his affected shoulder at night. A plain radiograph of the shoulder joint showed a punched-out eccentric bony erosion in the clavicle region of the right acromioclavicular joint and irregular opacity occupying the subacromiodeltoid bursa. MRI showed tophi deposits along the upper ridge of the distal end of the clavicle and in the subacromiodeltoid bursa [7]. Gouty arthritis of the shoulder joint, as described in the literature, is presented as tophaceous gout of the rotator cuff, causing subacromial impingement or rotator cuff tendinitis and shoulder dysfunction. In our case, the patient had no distinct appearance of acute arthritis, and there was severe bone destruction of the glenoid, resulting in severe dysfunction of the right shoulder joint. The gold standard for diagnosing gout is identifying characteristic monosodium urate crystals in the synovial fluid using polarized microscopy. During the patient’s diagnosis process, we considered the possibility of the patient suffering from gouty shoulder arthritis. Since a polarizing microscope was not available in our hospital, the diagnosis of gouty arthritis needed to be confirmed by clinical symptoms or histopathological analysis. The patient’s shoulder bone tissue was severely damaged, and at the same time, there was not a typical presentation of gouty arthritis. Therefore, we focused more attention on the possibility of shoulder neoplasia or infection. After diagnosing the case through histological examination, we carefully observed the patient’s imaging data. We found the typical features of gout: centralized erosions, sclerotic rim, and overhanging edges on the plain radiograph and CT.

In clinical work, gouty arthritis should be considered in patients who present with shoulder pain, weakness, and limited mobility, especially patients who have no distinct appearance of acute arthritis and have severe bone destruction of the shoulder joint. The differentiation of gouty arthritis from infectious arthritis or osteomyelitis is not always easy. It is necessary to exclude other possible etiologies, such as infection, neoplasia, and bone destructive disease. The following conditions must be considered in differential diagnosis: septic shoulder arthritis is associated with acute inflammation, redness, swelling, and pain; laboratory tests have a high percentage of white blood cells and neutrophils, and joint fluid examination will indicate microbes or pus cells. Tuberculous shoulder arthritis manifests as pain, dysfunction, muscular atrophy, and fistula; patients also have systemic symptoms of tuberculosis and are positive for the Xpert test [11]. Rapid destructive arthropathy of the shoulder joint is described as follows: the course of the disease develops rapidly; and there is bone destruction and tearing of the rotator cuff [12]. Milwaukee shoulder is a destructive calcium phosphate crystalline arthropathy related to the following factors: trauma or overuse, calcium pyrophosphate dehydrate crystal deposition, neuroarthropathy, dialysis arthropathy, denervation, female sex, and advanced age. The imaging findings are as follows: narrowing of the joint space, subchondral bone sclerosis with cystic changes, subchondral bone destruction, soft tissue swelling, calcification of the joint capsule, free bodies in the joint cavity, rotator cuff tears, and massive haemorrhagic joint effusion [13].

In this study, the patient’s shoulder joint pathology was insidious, and no apparent acute gout episodes occurred during the entire course of the disease; the condition mainly manifested as chronic arthritis, with right shoulder pain, weakness, and limited mobility. Although the patient had a history of gout, there had been no gout attacks in the shoulder joint; the attacks were mainly manifested in the bilateral feet. Additionally, it was found that the left shoulder joint was also affected by a gouty attack. However, there were no clinical symptoms, which caused some confusion and challenges regarding the diagnosis and treatment. The possibility of gouty arthritis was not initially considered. The patient had a long history of gout, with 2–3 attacks per year, mainly manifested in the bilateral feet, but there were no obvious gout attacks in other parts of the body. Only colchicine was taken orally during the gout attack periods to relieve the symptoms. The patient did not receive an effective and systematic treatment of gout. We believe that the severe destruction of the glenoid was related to an unhealthy diet and the lack of effective and systematic treatment. With effective therapy earlier in the disease process, severe bone destruction of the shoulder bone tissue can be avoided. This condition can lead to significant debilitation in patients if not identified early and managed appropriately.

Gouty arthritis involves the shoulder joint relatively rarely, and cases of osteolytic destruction of the bone tissue of the shoulder joint are rare. Gouty arthritis of the shoulder with severe bone destruction is easily misdiagnosed as a shoulder tumor or infectious disease. In conclusion, although gouty shoulder arthritis is considered unusual, when a patient has a history of gout, atypical manifestations, and severe erosive lesions of the glenoid in the shoulder joint, physicians and orthopedic surgeons should consider the possibility of gout causing severe lesions that mimic infection or neoplastic disease (Table 1). This case study aimed to alert physicians to the unusual manifestations and presentations of gouty arthritis, which could be missed if there is no suspicion.

**Table 1:** Key learning points

1. Gout can present as a mimicker of malignancies (metastasis) and septic arthritis.
2. Gout may affect many different articular and non-articular sites (e.g., musculoskeletal tissues, non-musculoskeletal tissues, and organs) [14].
3. Long-standing gout can present with atypical symptoms.
4. The manifestations of chronic shoulder gout are tophaceous deposits in the rotator cuff, intraosseous tophi in the bone, and tophi in the bursa around the shoulder joint. Gouty arthritis of the shoulder can result in severe erosive lesions of the bone tissues.

## Data Availability

The datasets used and/or analyzed during the current study are available from the corresponding author upon reasonable request.

## Abbreviations

CT: Computed tomography
MRI: Magnetic resonance imaging

## References

[1] Alqatari S, Visevic R, Marshall N, Ryan J, Murphy G (2018). An unexpected cause of sacroiliitis in a patient with gout and chronic psoriasis with inflammatory arthritis: a case report. BMC Musculoskelet Disord.

[2] Singh JA, Gaffo A (2020). Gout epidemiology and comorbidities. Semin Arthritis Rheum.

[3] Dehlin M, Jacobsson L, Roddy E (2020). Global epidemiology of gout: prevalence, incidence, treatment patterns and risk factors. Nat Rev Rheumatol.

[4] O’leary ST, Goldberg JA, Walsh WR (2003). Tophaceous gout of the rotator cuff: a case report. J Shoulder Elbow Surg.

[5] Chang CH, Lu CH, Yu CW, Wu MZ, Hsu CY, Shih TT (2008). Tophaceous gout of the rotator cuff. A case report. J Bone Joint Surg Am.

[6] Ichiseki T, Ueda S, Matsumoto T (2015). Rare coexistence of gouty and septic arthritis after arthroscopic rotator cuff repair: a case report. Int J Clin Exp Med.

[7] Tierra Rodriguez AM, Pantoja Zarza L, Brañanova López P, Diez Morrondo C (2019). Tophaceous gout of the shoulder joint. Reumatol Clin.

[8] Ragab G, Elshahaly M, Bardin T (2017). Gout: An old disease in new perspective - A review. J Adv Res.

[9] Gentili A (2006). The advanced imaging of gouty tophi. Curr Rheumatol Rep.

[10] McQueen FM, Doyle A, Dalbeth N (2011). Imaging in gout–what can we learn from MRI, CT, DECT and US. Arthritis Res Ther.

[11] Longo UG, Marinozzi A, Cazzato L, Rabitti C, Maffulli N, Denaro V (2011). Tuberculosis of the shoulder. J Shoulder Elbow Surg.

[12] Kekatpure AL, Sun JH, Sim GB, Chun JM, Jeon IH (2015). Rapidly destructive arthrosis of the shoulder joints: radiographic, magnetic resonance imaging, and histopathologic findings. J Shoulder Elbow Surg.

[13] Nadarajah CV, Weichert I. Milwaukee shoulder syndrome. Case Rep Rheumatol. 2014;2014:458708. 10.1155/2014/458708 PMC3914332 24551470

[14] Towiwat P, Chhana A, Dalbeth N (2019). The anatomical pathology of gout: a systematic literature review. BMC Musculoskelet Disord.

